# 5-hydroxymethylcytosine profiles in circulating cell-free DNA associate with disease status in patients with osteosarcoma

**DOI:** 10.21203/rs.3.rs-7199550/v1

**Published:** 2025-09-01

**Authors:** Evan W. Neczypor, Hailey Reisert, Kelley Moore, Elizabeth Zeldin, Robert A Dubin, Lauren Battle, Chuan He, Masanori Hayashi, Daniel A. Weiser, Mark A. Applebaum

**Affiliations:** The University of Chicago; Albert Einstein College of Medicine; The University of Chicago; Albert Einstein College of Medicine; Albert Einstein College of Medicine; Icahn School of Medicine at Mount Sinai; The University of Chicago; University of Colorado Anschutz Medical Campus, Children’s Hospital Colorado; Albert Einstein College of Medicine; The University of Chicago

## Abstract

5-hydroxymethylcytosine (5-hmC) is a marker of open chromatin and active gene expression. We profiled 5-hmC using plasma-derived cell-free DNA (cfDNA) from patients with osteosarcoma to assess its utility as a biomarker of disease status. Genes with differential 5-hmC levels were identified from a Discovery cohort consisting of patients with osteosarcoma and well children. An independent Validation cohort was evaluated using these signature genes. Hierarchal clustering using 262 osteosarcoma signature genes identified in the Discovery cohort identified two clusters of samples in the Validation cohort. Cluster 1 contained 10 of 12 samples from patients with primary disease or osseous metastases whereas Cluster 2 contained 26 of 33 samples from patients without active disease. Using a semi-quantitative osteosarcoma signature scoring system, the sensitivity and specificity to identify patients with active disease were 65% and 64%, respectively. This technique is feasible in this population and further investigation with larger patient cohorts is warranted.

## Introduction

Osteosarcoma is the most common malignant primary bone tumor in children and adolescents, with approximately 400 new pediatric diagnoses in the United States each year.^1^ Unfortunately, outcomes for osteosarcoma have not significantly improved since the introduction of multiagent chemotherapy.^2^ Despite more favorable outcomes for patients with localized disease, relapse is still frequent.^3,4^ Currently, surveillance methods lack the necessary sensitivity and specificity to adequately detect residual disease that can lead to recurrence or to rapidly identify those not responding to therapy. Beyond metastatic status, biomarkers to guide therapeutic decision making are limited. While percent necrosis from pathologic assessment of resected tumors following neoadjuvant chemotherapy is prognostic,^4^ efforts to intensify therapy based on this information have not improved outcomes.^5^ Amplification of the *MYC* proto-oncogene is also associated with greater risk of metastatic disease and poor prognosis,^6^ though molecular testing is not yet represented in clinical guidelines.^7^

Sarcomas are known to shed DNA into the blood.^8–11^ Several studies have evaluated circulating tumor DNA (ctDNA) fraction of total cell-free DNA (cfDNA) content obtained from the plasma of patients with osteosarcoma, and several have shown correlation with detection of ctDNA and outcome.^12–19^ However, these techniques have limited ability to identify novel regulatory networks that may contribute to disease progression. Furthermore, epigenetic approaches to understanding ctDNA have been limited in osteosarcoma.

The oxidation of methylated DNA cytosines to form 5-hydroxymethylation is the first step in the removal of methyl groups from DNA. The presence of 5-hydroxymethylcytosine (5-hmC) within promoters, gene bodies, and gene regulatory elements is associated with active gene expression.^20^ Through nano-hmC-seal technology, genome-wide 5-hmC analysis can be obtained from as little as 1ng of cfDNA.^21^ In neuroblastoma, these cfDNA-derived 5-hmC profiles have accurately identified metastatic burden and regulatory networks.^22–24^ We hypothesized that cfDNA-derived 5-hmC profiles obtained from patients with osteosarcoma would be distinct from healthy children and patients with neuroblastoma, associate with disease status, and identify biologically significant gene networks.

## Results

### Patient cohorts and generation of 5-hmC profiles.

The Discovery cohort consisted of five patients from the Children’s Hospital Colorado with osteosarcoma, all of whom had localized primary tumors without evidence of metastases (Table 2). Each patient in the Discovery cohort had a diagnostic sample that was used for signature discovery and additional samples (n = 16) at later timepoints (Table 3, **Supplementary Data 1**). The Validation cohort consisted of 17 patients from the Children’s Hospital at Montefiore with osteosarcoma (Table 2). A total of 73 blood samples were obtained from these 17 patients. Of these samples, 55 passed quality control and were included in the analysis. 18 samples were excluded due to poor sequence quality. In the final Validation cohort, there were nine samples from patients at diagnosis or prior to local control, thirteen samples from patients with residual or relapsed disease after local control, and 33 samples from patients with no evidence of disease on imaging who were either receiving post-local control adjuvant chemotherapy or were off therapy (Table 3, **Supplementary Data 1**). Twelve patients had serial samples collected. There were 95 samples from children with neuroblastoma of which 89 passed quality control. Disease burden of patients at the time of sample collection was high (n = 23), moderate (n = 12), low (n = 7), or none (n = 47).

### 5-hmC profiles from patients with osteosarcoma vary less than those from patients with neuroblastoma

**Figure 1:**
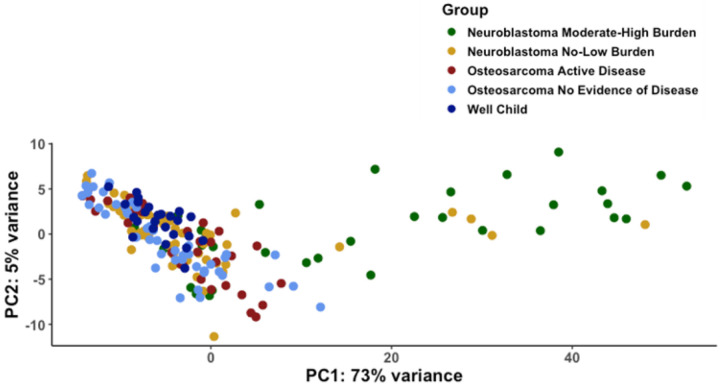
Principal Component analysis of whole genome 5-hmC demonstrates a high degree of variation among patients with neuroblastoma relative to patients with osteosarcoma and well children. PCA was performed with normalized 5-hmC counts of all genes adjusted for sex. Principal component 1 is strongly associated with presence of moderate-high burden neuroblastoma. Principal component 2 suggested differences between patients with active osteosarcoma and well children (p=0.069).

Principal component analysis (PCA) of all samples from patients with osteosarcoma, neuroblastoma, and well children showed that Principal Component 1 (PC1) effectively distinguished patients with moderate-high burden neuroblastoma from all other patient groups (Fig. 1, p = 3×10^−8^ via Kruskal-Wallis). There was no statistically significant difference in principal components among patients with active osteosarcoma, osteosarcoma patients with no evidence of disease, no or low burden neuroblastoma, or well children, suggesting that osteosarcoma derived ctDNA accounted for a relatively small percentage of cfDNA in all samples, as has been described.^14,15^

### cfDNA 5-hmC profiles correlate with disease status in osteosarcoma

Despite minimal variation among osteosarcoma samples relative to low/no burden neuroblastoma, principal component 2 suggested the presence of some differences between osteosarcoma patients and well children (p = 0.069 via Kruskal-Wallis). To further characterize this, we identified genes with elevated deposition of 5-hmC in osteosarcoma patients. We selected the 136 genes with elevated 5-hmC in the five diagnostic samples in the Discovery cohort compared to healthy children (top 10% fold-change, false discovery rate (FDR) < 0.05) as our osteosarcoma signature (**Supplementary Data 2**). We also identified 126 genes with significantly higher 5-hmC in well children (**Supplementary Data 2**).

**Figure 2:**
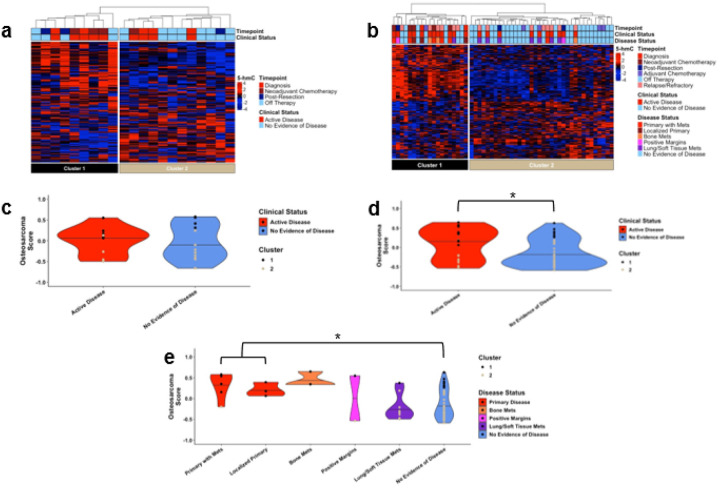
cfDNA 5-hmC profiles correlate with disease status. Unsupervised hierarchical clustering of osteosarcoma patient samples from the **a)** Discovery cohort and **b)** Validation cohort identified distinct clusters associated with disease status. The heatmaps were constructed using normalized 5hmC counts of 262 genes identified to have differential 5-hmC deposition on cfDNA of newly diagnosed osteosarcoma patients in the Discovery cohort compared to healthy control cfDNA. Violin plots of osteosarcoma signature scores by clinical status in **c)** the Discovery cohort and **d)** the Validation cohort, and **e)** by disease status in the Validation cohort. Horizontal lines are at the median score for each group. Individual samples represented as dots colored according to Cluster from hierarchal clustering in panels A and B. The gene set used in this analysis contained 136 genes identified to have increased 5-hmC in osteosarcoma patients versus healthy controls. *: p<0.05 by **d)**Wilcoxon test or **e)** Kruskal-Wallis test with post-hoc Dunn’s testing. Brackets between Primary with Mets and Localized Primary represent significance testing for these groups combined as Primary Disease.

Next, we performed unsupervised hierarchal clustering on both our Validation and Discovery cohorts using all 262 genes with differential 5-hmC deposition. Two distinct clusters were independently identified in both cohorts, defined as Cluster 1 and Cluster 2 (Fig. 2a–b). In both cohorts, Cluster 1 contained more samples from patients with active disease; 5 of 9 samples (55.6%) in the Discovery cohort and 12 of 19 (63.1%) in the Validation cohort. This latter Cluster contained 8 of 9 samples obtained prior to local control and 2 of 3 samples obtained from patients with osseous metastases. Conversely, in both cohorts, Cluster 2 contained more samples from patients without evidence of disease; 8 of 12 in the Discovery cohort (66.7%) and 26 of 33 (78.8%) in the Validation cohort. Of the eight samples from patients who only had lung (n = 7) and/or nodal metastases (n = 1), seven were in Cluster 2.

To quantitatively evaluate the presence of osteosarcoma derived cfDNA, we generated osteosarcoma signature scores for each sample using the 136 genes from our osteosarcoma signature. While there was no difference between samples from patients with active disease and those with no evidence of disease in the Discovery cohort (Fig. 2c, p = 0.9), samples from patients with active disease the Validation cohort had significantly higher scores than those with no evidence of disease (Fig. 2d, median 0.18 [IQR 0.67] versus − 0.26 [IQR 0.58], p = 0.025). This difference was primarily driven by patients with unresected primary tumors, who had significantly higher osteosarcoma scores compared to patients without radiologic evidence of disease (Fig. 2e, median 0.35 [IQR 0.38] versus − 0.26 [IQR 0.58], p = 0.035).

**Figure 3:**
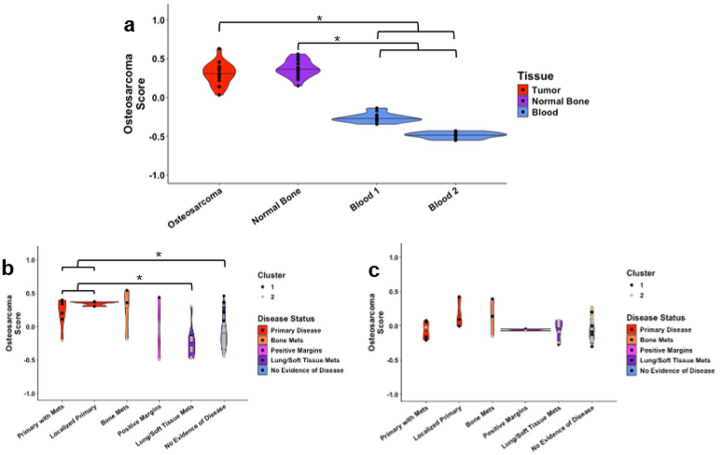
Validation of the biologic significance of osteosarcoma signature genes. Shown are violin plots of osteosarcoma signature scores. **a)** Relative RNA expression of 126 osteosarcoma signature genes identified in the Discovery cohort displayed according to tissue of origin from RNA sequencing data obtained from the gene expression omnibus. **b)**Relative 5-hmC deposition on 1,049 genes found to have increased RNA expression in osteosarcoma tissue versus peripheral blood from healthy donors displayed according to disease status in the Validation cohort. **c)** Relative 5-hmC deposition on 158 genes found to have increased RNA expression in osteosarcoma tissue versus matched normal bone displayed according to disease status in the Validation cohort. *: p<0.05 by Kruskall-Wallis test with post-hoc Dunn’s testing. Individual samples in panels b and c are represented as dots colored according to Cluster from Figure 2b.

We tested the tested the biologic soundness of our gene set by evaluating its performance on publicly available RNA sequencing data. RNA expression of our 5-hmC-derived osteosarcoma signature genes was significantly higher in osteosarcoma tumors and matched normal bone compared to peripheral blood from two independent cohorts of healthy controls (Fig. 3a, osteosarcoma median 0.30 [IQR 0.16] normal bone median 0.35 [IQR 0.12] versus blood median − 0.34 [IQR 0.21], p = 1.5×10^−10^). Next, we obtained genes with increased expression in osteosarcoma tumors compared to peripheral blood (1,049 genes, **Supplementary Data 3**). We used this gene set to perform Gene Set Variation Analysis (GSVA) scoring on our 5-hmC cfDNA samples. Scores were associated with disease status, similar to our osteosarcoma signature scores (Fig. 3b). These genes also overlapped significantly with our osteosarcoma signature genes (p = 0.003) by hypergeometric distribution testing. Furthermore, ontology terms enriched in both 5-hmC and RNA-derived gene sets included nervous system processes, GABA activity, and transmembrane signaling receptors (**Supplementary Data 4**). As GABA has been described as an activator of osteoblastic activity,^25,26^ these findings suggest that our blood-based osteosarcoma signature is likely reflective of abnormal bone or bone turnover. To further confirm this, we also identified 158 genes (**Supplementary Data 5)** with increased expression in osteosarcoma tumors compared to bone. Unlike our osteosarcoma signature, this gene set had no discriminatory power in the 5-hmC data (Fig. 3c).

Next, we sought to evaluate the specificity and sensitivity of osteosarcoma signature scores for detecting the presence or absence of disease among all patient samples in both cohorts. Defining a signature > 0 as detectable and < 0 as not detectable, the sensitivity and specificity to detect disease were 65% and 64%, respectively (**Supplementary Fig. 1**, AUC = 0.63 [95%CI = 0.50–0.76]). Inclusion of well children in the analysis improved specificity from 64–74% without affecting sensitivity (65%). Of the 25 well children, only two were false positives. Furthermore, patients with active disease had significantly higher scores than well children (**Supplementary Fig. 2, p** = 0.001). Fourteen of eighteen samples from patients with primary, unresected tumors and all three samples from patients with bony metastases were classified as having active disease and were considered true positives. Consistent with hierarchical clustering, samples from six of eight patients with isolated lung or nodal metastases were classified as having no active disease and were considered false negatives.

### MYC amplification can be detected by examining MYC 5-hmC in cfDNA

**Figure 4:**
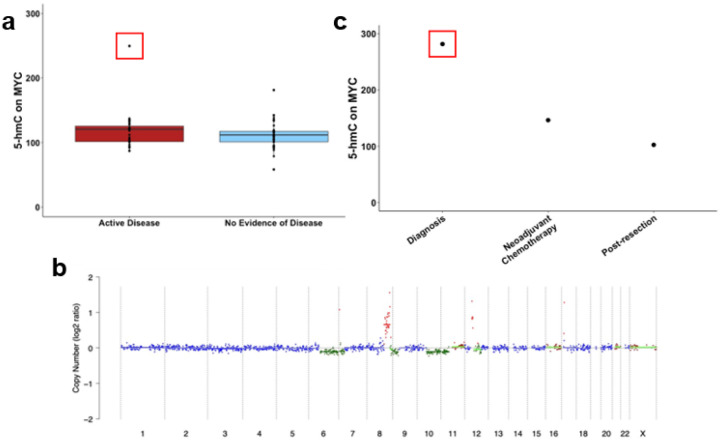
5-hmC levels on *MYC* demonstrate concordance with *MYC* amplification. **a)** Normalized 5-hmC counts of *MYC* among osteosarcoma patients with and without active disease on clinical imaging. Data point boxed in red indicates a diagnostic sample from a patient with confirmed *MYC* amplification. **b)** Copy number variation analysis of cfDNA from diagnostic patient sample with high *MYC* 5-hmC in panel A. ULP-WGS detected amplification of *MYC* on chromosome 8, represented as red dots. **c)**Normalized 5-hmC counts of *MYC* over the course of treatment of the patient identified to have *MYC* amplification.

We investigated normalized 5-hmC counts of *MYC* to determine if evidence of *MYC* amplification was detectable. While there was no difference in 5-hmC counts of *MYC* between patients with active disease and those with no active disease (p = 0.22), one sample had considerably higher 5-hmC deposition on *MYC* (Fig. 4a). This sample came from a patient at diagnosis within the Discovery cohort with confirmed *MYC* amplification (Fig. 4b). Additional samples from this patient during neoadjuvant chemotherapy and following resection of the primary tumor showed decreasing 5-hmC levels on *MYC*, consistent with decreased osteosarcoma derived cfDNA from a treated tumor and clinically decreased disease burden (Fig. 4c). There was also increased 5-hmC on *MYC* in one off therapy sample from a patient with *MYC* gain detected, and there were two additional samples with *MYC* amplification detected by ultra-low pass whole-genome sequencing (ULP-WGS) that were not evident in the 5-hmC data.

### cfDNA 5-hmC profiles change concordantly with disease status

We next sought to characterize changes in osteosarcoma signature scores in samples before and after surgery or the development of metastases. Nine patients had matched samples before and after surgery, and a decrease in signature score was observed in five following surgery (**Supplementary Fig. 3**, Mean change=−0.27 [SD 0.72]). Four of the five went from detectable osteosarcoma signature to undetectable (> 0 to < 0). Additionally, there were two patients with samples collected after resection with positive margins. Of these samples, one had a high osteosarcoma score (0.55) and was in Cluster 1 (Fig. 2b). The resected tumor had 15% necrosis, and the patient ultimately died of disease. The other sample from a patient with incomplete resection had a low osteosarcoma score (−0.54) and was in Cluster 2 (Fig. 2b). The resected tumor had 95% necrosis, and the patient remains in remission. Samples were available for two patients who developed osseous metastases before and after detection by imaging. In both patients, osteosarcoma scores increased at the time of detection of the metastases (average increase = 0.86 SD = 0.19), and both went from undetectable to detectable.

## Discussion

Utilizing Nano-hmC-Seal technology to map whole genome 5-hmC in circulating cfDNA, we showed that 5-hmC derived signatures of osteosarcoma are detectable in patients with active disease and are reflective of active bone turnover. Specifically, patients with primary tumors and osseous metastases had detectable osteosarcoma signatures while those with lung/nodal metastases did not. There were also data suggestive of feasibility for identifying *MYC* aberrant tumors and changes in clinical disease burden after surgery or at relapse. Consistent detection and monitoring of ctDNA in osteosarcoma has proven challenging, and an epigenomic approach incorporating 5-hmC analysis may provide complementary information to other methods.

This is the first study to evaluate 5-hmC profiling of cfDNA among patients with osteosarcoma and to directly compare cfDNA profiles between osteosarcoma and other pediatric solid tumors. Compared to neuroblastoma, 5-hmC profiles from patients with osteosarcoma varied less from well children and off therapy patients, suggesting that ctDNA may be less readily identifiable in patients with osteosarcoma. This is consistent with prior studies which have shown higher percentages of the ctDNA fraction at diagnosis in neuroblastoma (60%, range 3–99%, 100% detection)^27^ compared to osteosarcoma (11%, range 5–58%, 57% detection).^15^ The challenges in detecting osteosarcoma ctDNA have been reflected in several studies with inconsistent ctDNA detection among patients with active disease.^14,16–18^

5-hmC signatures distinguished patients with active disease from those with no evidence of disease with a sensitivity of 65% and specificity 64%. This performance is comparable to a previous study by Barris et al. which used targeted deep sequencing and obtained a sensitivity and specificity of 50% and 44%, respectively.^14^ Another study was able to predict relapse with a sensitivity and specificity of 67% and 95% using tumor-informed minimum residual disease panels of SNVs.^18^ In the present study, osteosarcoma scores decreased following surgery for local control of tumors, and we identified multiple patients with increased osteosarcoma scores at relapse or relapse progression. Furthermore, we were able to track *MYC* 5-hmC levels in a patient with confirmed *MYC* amplification, and we found their *MYC* levels decreased following chemotherapy and resection. ctDNA dynamics and molecular features over time remains understudied and will require increased numbers of samples from patients treated uniformly on clinical trials.

As 5-hmC deposition usually correlates with active gene transcription, we evaluated the potential biologic significance of the gene set. The osteosarcoma signature was highly expressed in osteosarcoma tissue and normal bone compared to peripheral blood. The signature also overlapped significantly with genes highly expressed in osteosarcoma tumors. Several of the overlapping ontology terms were related to GABA receptors, and GABA activity has previously been associated with human bone formation.^25,26^ These data suggest that the signature may be reflective of expression patterns of bone which may contribute to circulating DNA in the setting of rapid cell turnover within osseous tumors.

cfDNA 5-hmC profiles from patients with lung/nodal disease were similar to those of patients without evidence of disease. There are several possible explanations for this finding. Primary tumors are typically an order of magnitude larger than metastases (cm versus mm) and likely shed more ctDNA.^28,29^ Further, soft tissue metastases may have epigenetic programs that differ from primary tumors and osseous metastases. Larger cohorts with more patients with relapsed disease are needed to determine if alternative gene sets can identify patients with small lung or nodal metastases.

There are several limitations to these analyses. We were unable to assess whether 5-hmC profiles at diagnosis were associated with clinical outcomes, as there were limited numbers of samples from newly diagnosed patients. Additional diagnostic samples or tissue from tumor biopsies would also likely allow for refinement of osteosarcoma signature genes, leading to improved specificity and sensitivities to detect active disease. We are yet to establish the lower limit of detection for the osteosarcoma signature and have not directly compared 5-hmC data to other methods of detecting ctDNA and circulating tumor cells.^30^ Further studies will be needed to understand how different assays can complement each other. We are also unable to make conclusions about the ability for 5-hmC cfDNA profiling to detect minimal residual disease prior to detection on imaging, as only one sample was obtained from a patient who was off therapy prior to relapse.

In conclusion, this study found that osteosarcoma 5-hmC profiles can be detected in patients with osteosarcoma. Utilization of 5-hmC analysis has comparable sensitivity and specificity to detect osteosarcoma-derived cfDNA and may be complementary to other methods. Combining 5-hmC analysis with other approaches may improve the performance of cfDNA analysis for patients with osteosarcoma. Additional studies in larger patient cohorts are warranted.

## Methods

### Patients and samples

Peripheral blood samples were obtained from patients diagnosed with osteosarcoma between birth and 34 years old from the Children’s Hospital at Montefiore and the Children’s Hospital Colorado between May 2016 through December 2019. Well child controls and samples from patients with neuroblastoma were collected as described.^22^ Protocols were approved by the local IRBs and informed consent was obtained from all patients or their guardians.

For patients with osteosarcoma, blood was collected at diagnosis, during neoadjuvant therapy, post-resection (within one week of resection), during adjuvant chemotherapy, off therapy, and during relapse/refractory disease. Clinical status at the time of blood collection was dichotomized as either detected or no evidence of disease according to clinical imaging. Disease status at the time of blood collection was defined according to specific clinical criteria (Table 1). Among patients with neuroblastoma, metastatic disease burden was classified as described.^22^

### Blood collection and isolation of cfDNA

For all samples, 6–10 ml of blood was collected in EDTA tubes. All blood samples were stored at 4°C and processed within two hours of collection as described.^22^ cfDNA was isolated from 1–2 ml of plasma using the QIAamp Circulating Nucleic Acid Kit (Qiagen, Gaithersburg, MD) per manufacturer’s instructions with a final elution using 25 μl of IDTE pH 8.0 buffer.

### Nano-hmC-Seal library preparation and sequencing

Nano-hmC-Seal libraries were constructed from 1–10 ng of cfDNA as described.^20^ Briefly, after ligation with sequencing adapters using the KAPA Hyper Prep Kit (KAPA Biosciences), cfDNA 5-hmC marks were subjected to T4 beta-glucosyltransferase enzymatic modification with UDP-N_3_-glucose, followed by subsequent chemical modification with biotin-PEG4-dibenzocyclooctyne (DBCO). Biotin-labeled cfDNA fragments were then pulled down using streptavidin M270 Dynabeads (Invitrogen), and PCR amplified to construct sequencing libraries which were purified with AMPure XP beads (Beckman Coulter) to construct libraries.

Fifty base-pair, paired-end libraries were sequenced on an Illumina NextSeq 500. FastQC v0.11.7 was used to assess sequence quality (https://www.bioinformatics.babraham.ac.uk/projects/fastqc/). Raw reads were processed with Trimmomatic v0.36.^31^ Remaining reads were aligned to hg38 with Bowtie2 v2.3.4.3 using default settings.^32^ Duplicated reads were removed using Picard v2.18.29 (https://gatk.broadinstitute.org/hc/en-us/articles/360037052812-MarkDuplicates-Picard). Aligned reads were counted using featureCounts of Subread v1.5.3^33^ using the gene flag and the gencode.v33.annotation.gtf file from GENCODE.^34^ Samples were retained if they passed quality control of library construction, fragment analysis, and principal component analysis.

### Identification of circulating osteosarcoma DNA in cfDNA samples

Read counts of 5-hmC across the entire gene body were loaded into DEseq2 v1.40.2^35^ package in R v4.3.0 with a differential 5-hmC model adjusted for sex. Principal component analysis of all samples was performed using BiocGenerics v0.46.0.^36^ Genes with differential 5-hmC in patients with osteosarcoma compared to well children were identified using an FDR < 0.05 and 10% greatest fold change. ComplexHeatmap v2.16.0 was used to perform hierarchal clustering based on cfDNA 5-hmC deposition in patient samples.^37^ g:Profiler^38^ was used to conduct gene set enrichment analysis of significant genes.

Gene Set Variation Analysis (GSVA) v1.48.3^39^ was used to quantify osteosarcoma signature scores. GSVA is a non-parametric, unsupervised gene set enrichment analysis method that returns values from − 1 to 1, with more positive values indicating more enrichment to a reference gene set. 5-hmC counts per gene were normalized and adjusted for sex and batch prior to GSVA scoring. Area under the receiver operator characteristic analyses of GSVA scores for identifying active disease were implemented using the pROC package v1.18.5.^40^

### Analysis and application of publicly available RNA sequencing data

RNA sequencing read counts were obtained from osteosarcoma tumors, patient-matched normal bone biopsies (GSE99671),^41^ and whole blood of healthy controls (GSE112057, GSE164890).^42,43^ Genes with differential expression were identified as above. GSVA analysis was used to score samples as above.

### Analysis of MYC amplification

Normalized counts of 5-hmC on *MYC* were obtained for all samples from patients with osteosarcoma in both cohorts. To evaluate for *MYC* amplification, ultra-low pass whole-genome sequencing (~ 0.5x) was performed on an Illumina HiSeq 2500. We applied the ichorCNA algorithm as described.^44^ In samples with circulating tumor DNA detected, copy number alterations in the region of *MYC* at chromosome arm 8q were classified as gain (up to 3 copies) or amplified (4 or more copies).

### Statistical Analyses

To compare GSVA scores, either a two-sample Wilcoxon test or a Kruskal-Wallis rank sum test with post-hoc Dunn’s pairwise multiple comparison testing using Benjamini-Hochberg correction were performed with an adjusted p-value < 0.05 considered significant. Enrichment of various biologic processes and diseases were considered significant with an FDR < 0.05.

## Supplementary Material

Supplementary Files

This is a list of supplementary files associated with this preprint. Click to download.

- SupplementalFiguresnpj.pdf
- SupplementalDatanpj.xlsx

## Figures and Tables

**Table 1:** Classification of disease status for samples. Description of criteria used to assign disease status to each patient sample obtained in the Validation cohort.

Disease Status	Criteria
Primary with Mets	Unresected primary tumor with metastases present on imaging. Samples obtained at diagnosis, neoadjuvant chemotherapy, refractory disease.
Localized Primary	Unresected primary tumor without metastases present on imaging. Samples obtained at diagnosis or during neoadjuvant chemotherapy
Bone Mets	Evidence of osseous metastases with or without lung/soft tissue metastases on imaging after complete resection of primary tumor
Lung/Soft Tissue Mets	Evidence of lung/soft tissue metastases without osseous metastases on imaging after complete resection of primary tumor.
Positive Margins	Sample collected within 1 week of primary tumor resection with positive margins on pathologic review. No presence of metastatic disease on imaging.
No Evidence of Disease	No evidence of disease on clinical imaging or pathologic review after full resection of primary tumor. Samples collected from patients off therapy or during adjuvant chemotherapy.

**Table 2:** Patient characteristics of the Discovery and Validation cohorts. NA: not applicable, SD: standard deviation.

Feature	Discovery cohort (n = 5)	Validation cohort (n = 17)
**Age at Diagnosis** (median, SD)	13.0, 2.7	14.6, 6.4
**Sex, No. (%)**		
Male	1 (20%)	9 (53%)
Female	4 (80%)	8 (47%)
**Race, No (%)**		
White	5 (100%)	4 (24%)
Black	0	4 (24%)
Asian	0	0
Other	0	6 (35%)
Unknown	0	3 (18%)
**Ethnicity, No (%)**		
Non-Hispanic	4 (80%)	6 (35%)
Hispanic	1 (20%)	7 (41%)
Unknown	0	4 (24%)
**% Necrosis**		
≥ 90%	2 (40%)	4 (24%)
< 90%	2 (40%)	11 (64%)
No Resection/Unavailable	1 (20%)	2 (12%)
**Outcome**		
Free of Disease	4 (80%)	8 (47%)
Relapsed/Refractory, Alive	0	2 (12%)
Dead of Disease	1 (20%)	7 (41%)
**Primary Tumor Site**		
Lower Extremity	4 (80%)	9 (53%)
Upper Extremity	0	1 (6%)
Sacrum	0	1 (6%)
Rib	0	1 (6%)
Scapula	0	1 (6%)
Spine	1 (20%)	0
Skull	0	1 (6%)
Multifocal	0	3 (17%)
**Primary tumor size** (median, SD)	44 mm, 18 mm (n = 5)	108mm, 34mm (n=4)
**Bone metastases size** (median, SD)	NA	29mm, 1mm (n = 2)
**Lung metastases size** (median, SD)	NA	6mm, 10mm (n = 7)
**Nodal metastases size** (median, SD)	NA	43mm, 39mm (n = 2)

**Table 3:** Clinical correlative information for samples from the Discovery and Validation cohorts.

Feature	Discovery cohort (n = 21)	Validation cohort (n = 55)
**Sample Collection Time, No. (%)**		
Diagnosis	5 (24%)	2 (4%)
Neoadjuvant Chemotherapy	4 (19%)	5 (9%)
Post-Resection of Primary Tumor	3 (14%)	3 (6%)
Adjuvant Chemotherapy	0	5 (9%)
Off-Therapy	8 (38%)	26 (47%)
Relapse/Refractory	1 (5%)	14 (25%)
**Clinical status at sample collection, No. (%)**		
Active Disease	10 (48%)	22 (40%)
No Evidence of Disease	11 (52%)	33 (60%)
**Disease status at sample collection, No. (%)**		
Primary with Mets	0	6 (11%)
Localized Primary	9 (43%)	3 (5%)
Bone Mets	0	3 (5%)
Lung/Soft Tissue Mets	0	8 (15%)
Positive Margins	0	2 (4%)
No Evidence of Disease	12 (57%)	33 (60%)
**Metastases Present on Imaging at sample collection, No. (%)**		
Yes	0	17 (31%)
No	21 (100%)	38 (69%)

## Data Availability

The raw 5-hmC sequencing data used in this study are available through the NCBI dbGaP database under accession number phs003842.v1.p1, titled “Feasibility of Circulating 5-hydroxymethylcytosine profiling in Osteosarcoma.”, and phs001831.v1.p1, titled “Elucidating Transcription Regulation by Epigenetics in Neuroblastoma.”

## Code Availability:

The underlying code for this study is not publicly available but may be made available to qualified researchers on reasonable request from the corresponding author.
